# Healthcare workers' attitudes towards working during pandemic influenza: A multi method study

**DOI:** 10.1186/1471-2458-8-192

**Published:** 2008-06-02

**Authors:** Heather Draper, Sue Wilson, Jonathan Ives, Christine Gratus, Sheila Greenfield, Jayne Parry, Judith Petts, Tom Sorell

**Affiliations:** 1 Centre for Biomedical Ethics, Department of Primary Care and General Practice, The University of Birmingham, Edgbaston, Birmingham, B15 2TT, UK

## Abstract

**Background:**

Healthcare workers (HCWs) will be key players in any response to pandemic influenza, and will be in the front line of exposure to infection. Responding effectively to a pandemic relies on the majority of medical, nursing, laboratory and hotel services staff continuing to work normally. Planning assumes that during a pandemic normal healthcare service levels will be provided, although it anticipates that as caseloads increase only essential care will be provided. The ability of the NHS to provide expected service levels is entirely dependent upon HCWs continuing to work as normal.

**Methods/design:**

This study is designed as a two-phase multi-method study, incorporating focus groups and a questionnaire survey. In phase one, qualitative methods will be used to collect the views of a purposive sample of HCWs, to determine the range of factors associated with their responses to the prospect of working through pandemic influenza. In phase two, the findings from the focus groups, combined with the available literature, will be used to inform the design of a survey to determine the generalisability of these factors, enabling the estimation of the likely proportion of HCWs affected by each factor, and how likely it is that they would be willing and/or able to continue to work during an influenza pandemic.

**Discussion:**

There are potentially greater than normal health risks for some healthcare workers working during a pandemic, and these workers may be concerned about infecting family members/friends. HCWs will be as liable as other workers to care for sick family members and friends. It is vital to have information about how motivated HCWs will be to continue to work during such a crisis, and what factors might influence their decision to work/not to work. Through the identification and subsequent management of these factors it may be possible to implement strategies that will alleviate the concerns and fears of HCWs and remove potential barriers to working.

## Background

The World Health Organisation (WHO) describes an influenza pandemic as an event in which "a new influenza virus appears against which the human population has no immunity, resulting in several, simultaneous epidemics worldwide with enormous numbers of deaths and illness" [1] Estimates of the impact of such an event vary, but the WHO offers this guidence:

"today a pandemic is likely to result in 2 to 7.4 million deaths globally. In high income countries alone, accounting for 15% of the world's population, models project a demand for 134–233 million outpatient visits and 1.5–5.2 million hospital admissions. However, the impact of the next pandemic is likely to be the greatest in low income countries because of different population characteristics and the already strained health care resources" [1].

Despite the UK being a developed and relatively affluent country, the effects of an influenza pandemic are likely to be considerable. It is impossible to predict the exact impact of the next pandemic, but Department of Health (DH) estimates suggest that up to half the UK population could become infected (30 million people), with between 50,000 and 750,000 additional deaths as a result [2]. DH planning assumptions work on the basis of cumulative clinical attack rates of up to 50%, with 4% of symptomatic patients requiring hospital admission (1,200,000 people). A case fatality rate of between 0.2 and 2.5% is assumed, based on previous pandemics [2]. Even at the lower end of these estimates, an influenza pandemic will place the UK healthcare system under severe strain.

Healthcare workers (HCWs) will be key players in any response to pandemic influenza, and will be in the front line of exposure to infection. Responding effectively to a pandemic relies on the majority of National Health Service (NHS) staff continuing to work normally. Planning assumes that during the pre-pandemic phase 'normal' healthcare service levels will be provided in the UK. Once the outbreak reaches the UK, and a pandemic is confirmed, the NHS "plans to care for large numbers of cases, and will only provide essential care" p31 [2]. Staff absenteeism is estimated to be around double that of normal, including those who have to care for sick family members [3]. The ability of the NHS to provide even basic essential care is entirely dependent upon the remaining HCWs continuing to work as normal.

Planners cannot assume that HCWs will be willing to work normally even if they are able to do so. For instance, during the early years of the Human Immunodeficiency Virus (HIV) epidemic doctors debated whether it was acceptable to refuse to treat those with HIV [4-8]; and during the Severe Acute Respiratory Syndrome (SARS) outbreak some HCWs were not willing to treat SARS patients [9,10]. It is debateable whether professionals have a duty to work normally during a pandemic or other emergency [9,11], and it is also debatable to what extent this duty could or should be enforced if it exists.

There is limited data on factors that may influence normal working, including prior professional obligation, personal risk and risk to families of HCWs, and inclusion in preparedness planning [12-14]. In Germany, Ehrenstein *et al *[15] found 28% of professionals (clinical and non-clinical) may abandon work in favour of protecting theselves and their family. Quereshi *et al *[16] found that the most significant barrier to HCWs' willingness to work was fear for their own and their families' health. Based on a survey of mixed clinical and non-clinical workers in the USA, Balicer *et al *[17] anticipate up to 50% of HCWs being unwilling to work, with clinical staff more likely to attend than non-clinical ones. These studies may have limited applicability to the UK and provide insufficient information to inform attempts to modify attitudes ahead of a pandemic.

Professional codes of conduct do not insist on normal working when there is personal risk [9], and norms of professional ethics may have to be suspended during a pandemic, which could be an additional source of stress for HCWs. For example, scarcity of resources and staff shortages may lead to the standards of care having to drop below what would be acceptable in 'normal' times. Staff may have to refuse care to people whom they could ordinarily easily help. Normal, but time consuming, consent procedures may have to be abandoned. All of these may well be ethically justified in an emergency situation, but nonetheless may be distressing for HCWs. Trust in government and management may affect HCWs' perceptions and reactions to a pandemic for two reasons. First, trust that the pandemic is being well-managed and that information they are provided with is reliable might reassure individuals that the work they are being asked to do is an effective way of dealing with the pandemic [18]. Second, people's attitudes towards taking additional risks might be affected by their perceptions of the extent to which these additional risks are attributable to action or inaction on the part of their managers/the government [19,20].

Contingency planning, and consequently patient care, will be improved if it is possible to predict the factors likely to affect UK HCWs' willingness and ability to work, and to identify the motivations HCWs have for continuing to work. By helping to identify the characteristics of those who may be unwilling to work, the study will inform assignments of staff to pandemic work, and suggest options for changing the attitudes of those who might be unwilling. The study may also identify types of provision (e.g. housing at hospitals for HCWs) that would keep pools of nearby staff high, resolve travel problems and limit risks to workers' families. If attitudes identified as prevalent are based on misconceptions, an information campaign for HCWs could be mounted to address these, helping to ensure higher attendance for duties than had misconceptions remained in place.

Of particular importance to this study is the motivation of HCWs during pandemics. Recently, conventional focus group methodology has been adapted to tease out participants' justifications for the views they hold [21-23]. Rather than simply registering attitudes, this methodology challenges the views of participants and encourages them to articulate their arguments and justifications. This strategy will be useful in determining the strength and basis of HCWs' sense that they have or lack an obligation to work normally.

The primary aim of this research is to enhance contingency planning and national preparedness by:

• Exploring HCWs' views about their obligations to continue working during an influenza pandemic.

• Determining what factors might positively or negatively affect HCWs' willingness to continue working during an influenza pandemic.

• Determining what measures might enable or help HCWs to continue working during an influenza pandemic.

• Estimating the proportion and characteristics (professional/non-professional, age, sex, family commitments etc.) of HCWs likely to continue working during an influenza pandemic.

The secondary aims are:

• To determine factors related to pandemic influenza that may erode an HCWs' sense of professional obligation.

• To explore how much HCWs know about pandemic influenza and the measures affecting them that may be considered necessary during a pandemic.

## Methods/design

This study is designed as a two-phase multi-method study, incorporating focus groups and a questionnaire survey. In phase one, qualitative methods will be used to collect the views of a purposive sample of HCWs, to determine the range of factors associated with their responses to the prospect of working through pandemic influenza. In phase two, the findings from the focus groups, combined with the available literature, will be used to inform the design of a survey to determine the generalisability of these factors, enabling the estimation of the likely proportion of HCWs affected by each factor, and how likely it is that they would be willing and/or able to continue to work during an influenza pandemic.

The study setting is the West Midlands region of the United Kingdom, which comprises 10% of the population of England, and is demographically comparable (age, gender) to the wider population, with relatively high proportions of ethnic minorities in some areas (24). Within the West Midlands, healthcare services are provided in both rural and urban settings, and in teaching/non-teaching hospitals/community healthcare practices, allowing access to a wide range of HCWs across a wide demographic range.

### Phase 1: Qualitative study

Focus groups, lasting around 90 minutes, will explore attitudes to working during an influenza pandemic. The topic guide will use broad themes in order to establish a wide framework for discussion; for example: What inclines/disinclines participants to come to work? Do participants perceive work as a duty? How is the requirement to work balanced against family demands?

The aim will be to encourage participants to discuss views about their own, and other HCWs', obligations to stay at post during a pandemic and to try to isolate and articulate the source of any sense of obligation or sense that there is no obligation (e.g. professionalism, religious beliefs, not wanting to let colleagues down, being part of a team, financial pressures, family commitments, self-preservation, low job satisfaction, poor departmental morale etc.), what concerns them about staying at post (e.g. perceived vs. actual risk of infection, longer working hours, understanding of dangers), and what could be done to alleviate these concerns. In order to ensure that the discussions are participant led, and not influenced by the themes expected by the researchers, a topic guide will be used which asks a series of open questions:

1. What do you know about pandemic influenza?

2. Have you had any training in the management of pandemic influenza? Do you feel this training has been sufficient?

3. What effect do you think pandemic influenza might have on your workplace?

4. UK contingency planning favours a message of 'business as usual' as far as possible during pandemic influenza. How likely do you think it is that it will be 'business as usual' for you?

4.1 If you were fit and well during any influenza pandemic, how likely do you think it is that you would carry on working?

4.2 Some people think that healthcare workers have unconditional obligations to work, even when the risks to themselves are great, but others disagree. What do you think?

5. What are the kinds of things that might worry you about working during a pandemic?

6. What kinds of things do you think could be done by the Department of Health/your employing NHS Trust/Professional bodies/Trade Unions to ease those worries?

Where it is not possible to run focus groups, for example, because of the difficulty of getting together senior medical staff for a 90 minute focus group, semi-structured interviews will be conducted, using the same topic guide as the focus groups.

#### Facilitation and Analysis

Initial responses to these open questions will be probed and explored by the facilitator, who will ask more specific questions as and when appropriate. Facillitation of focus groups and interviews will be directed towards eliciting participants' moral reasoning and justifications for the claims they make about their own, and HCWs' in general, obligations to work during an influenza pandemic. Where necessary during the focus groups, and as a matter of course during interviews, the facilitator will make use of counter examples and counterfactual cases to challenge particpant's claims, and will put forward contrary or alternative views emerging from prior groups/interviews in order to tease out the points of disagreement and the variety of motivations that may be at work.

Focus groups and interviews will be digitally recorded and then transcribed *ad verbatim*. Hand-written notes will be taken as a back up to this process and to record non-verbal interactions.

Constant comparative analysis will be utilised, with data collection and analysis occuring simultaneously. Emerging themes relevant to the research questions will be identified and fed back into subsequent groups and developed as an iterative process in order to generate theories to explain HCWs' willingness to work. Detailed qualitative analysis of recordings will generate a thematic framework that identifies for each individual focus group and interviewee their knowledge about pandemic influenza, their ideas about work and their sense of obligation to work, and the factors that act to influence their commitment to go to work during an influenza pandemic. The emerging themes will be used to inform the design of the postal/online survey in phase two.

The analysis will be conducted both by specialists in qualitative resesarch and medical sociology and by specialists in public health and medical ethics, allowing a thorough analysis of emerging themes alongside an analysis of the moral reasoning used to justify participants' claims. Content analysis will identify recurring themes relating to HCWs' willingness and ability to work. The content analysis will also focus on normative themes – i.e. the reasons given by participants to justify the claims they make about the duty (or lack of) to work during an influenza pandemic – through the lens of moral theory (identifying different kinds of moral reasoning as consequentialist, deontological, virtue based etc.).

#### Recruitment

Ten focus groups will be run, each with 6–8 participants, across the range of HCWs likely to be affected during a pandemic. Purposive sampling will be used. The composition of these groups also has to accommodate the inhibitions individuals might feel in speaking freely if group members cross the hierarchical structures of working life. The following groups were therefore identified:

1 × hospital doctors across specialties (consultants)

1 × hospital doctors across specialties (non-consultants)

1 × General Practitioners (GPs).

1 × managers – including GP practice managers and public health doctors.

2 × nurses across specialities and grades – including students, health care assistants (HCAs) and midwives.

2 × ancillary staff – porters, mortuary attendants, switchboard, cleaners, laundry etc.

1 × community based HCWs – practice nurses, community midwives, health visitors.

1 × professionals allied to medicine – occupational therapists, radiographers, pharmacists, laboratory workers, phlebotomists, ambulance staff etc.

We will identify two NHS Acute Hospital Trusts in the West Midlands from which participants will be recruited. One of these will contain a large teaching hospital in an inner city area and the other will be a District General Hospital with a more rural catchment. Trusts will identify potential participants and letters of invitation will be sent, with a participant information sheet, asking those who are interested in participating in the focus groups to contact the research team. Posters asking for volunteers for the focus groups will also be placed in staff areas inviting those interested to contact the research team. Potential participants will be able to phone, text or e-mail for more information. GPs and Community Healthcare workers will be recruited through letters sent to general practices inviting participation.

#### Consent

Written consent to participate in the focus group/interview will be taken by members of the research team as participants arrive. This will include a declaration that the participants will not talk about each other's comments outside of the meeting – to remind those involved to respect the privacy of others and to encourage frankness. All particpants will have received a participant information sheet when they were invited to participate. Prior to participation all participants will have been in contact with a member of the research team and will have been invited to discuss their participation, including any questions they may have.

### Phase 2: Questionnaire survey

In this phase a survey of approximately 3,000 HCWs will be carried out to determine the factors significantly associated with a decision to continue to work and the proportion and characteristics of people perceiving they are likely to continue to work. A questionnaire will be sent to HCWs employed in the West Midlands, alongside instructions to access an online version if this is preferred. In addition, the survey will be available for completion online by uninvited participants. Invited and uninvited participants will be distinguished by a unique identifier, which invited particpants will be asked to enter on the online survey.

The questionnaire content will be informed by the available literature and the findings from phase one, and will include socio-demographic factors (e.g. age, gender, country of birth), category of HCW (occupational group, tenure, or length of service with, line manager or non-line manager, full time or part time, shift worker or non-shift worker), knowledge of pandemic influenza, home circumstances (e.g. carer to children/parents), distance from home to work, perceived risks and benefits of continuing to work, likelihood of continuing to work in different circumstances, agreement/disagreement with statements of various ethical principles.

Closed questions using validated response categories and rating scales to determine attitudes and preferences will be used. Confidentiality of responses will be assured. Questionniares will be marked with a unique identifier and one reminder will be sent to all non-responders. Freepost envelopes will be provided. Anyone who wishes to participate but who experiences difficulties completing the paper or online questionnaire will be offered the opportunity of completing it over the telephone or face-to-face with one of the research team.

#### Bias and confounding

Piloting will ensure readability and acceptability, alongside review from the project steering committee. Estimates of responses by age, sex and deprivation score will be made and standardised rates calculated to address response bias and allow for the fact that we will purposively aim to secure at least 150 responses from each category of HCW.

#### Sample size estimates

Of the 1.3 million staff in the NHS, over 80% (1.145 million) are front line staff, with the remainder (220,000) providing infrastructure support. Of the latter, nearly one-half (106,000; 48%) are in central functions, just over one-third (75,000; 34%) in hotel, property and estates and just under one-fifth (39,000;18%) are managers. Sixty percent (679,000) of front-line employees are professionally qualified clinical staff (over 122,000 doctors and 404,000 nurses), the remainder comprising non-clinical support staff.

Assuming an average Trust has a staff of 1500, recruiting 10 Trusts will generate a sample of 15000 individuals. Our previous surveys of healthcare professionals have achieved response rates of the order of 50–60% [25-28]. Higher response rates are associated with short questionnaires and where the topic is of interest to the individual [29]. We anticipate that pandemic influenza will be a topic of considerable interest but, nevertheless, we have assumed a very conservative 25% response rate, an estimated 3,750 responses. This will be sufficient to determine the overall proportion with a positive attitude to continuing working with an overall precision of 2% (95% confidence), when a worst case assumption of 50% with a positive attitude to continuing working is used. Should this percentage be greater than (or less than) 50%, precision will be improved. Targeted approaches will be used to ensure that a minimum of 150 responses have been achieved from each of the 10 categories of HCWs, this will ensure that the percentage with a positive attitude to continuing working can be estimated with 95% confidence and 8% precision in each sub-group and to demonstrate differences of the order of 16% between sub-groups of HCWs with 80% power at the 5% significance level.

#### Recruitment

The target population will comprise all HCWs (e.g. hospital doctors, public health doctors, GPs, community nurses, healthcare managers, nurses, allied health professionals, paramedics, ancillary ward staff, porters, laboratory staff, hospital laundry workers and hospital cleaners). Healthcare Trusts will be purposively recruited to ensure the inclusion of different groups (urban/rural, affluent/deprived, primary care/District General Hospital (DGH)/Acute Trust/tertiary referral centre).

Participating Trusts will be asked to identify potential participants based on sampling information provided by the researchers. An invitation letter, and a copy of the questionnanire, will be distributed to staff via the internal post or Trust email according to local preferences. Participants will have the choice of completing a paper or on-line questionnaire.

Targeted approaches will be used for those sub-groups who do not routinely use Trust post/email (e.g. contract cleaners) and staff coffee rooms and meetings used to access any under-represented groups (e.g. gender, seniority). Our aim will be to ensure a minimum of 150 responses from each of the 10 groups of HCWs (defined above for the focus groups). Structured telephone interviews (following the format of the questionnaire) will be offered to those with poor literacy levels or visual impairments. A large print version of the questionnniare will be available on request.

#### Consent

Formal consent will not be taken, and completion of the questionnaire will be considered evidence of consent. Potential participants will have received information about the questionnaire and the project at the time of the invitation.

#### Analysis

Descriptive analyses will concentrate on the ratings given to the decision to continue to work, including the percentage with a positive attitude, and the characteristics of those giving low and high ratings to continuing to work during a pandemic. Associations between attitudes to continued working, agreement/disagreement with ethical principles, category of HCW and demographic characteristics will be explored using non-parametric statistics. Combinations of characteristics best describing individuals with differing intentions to continue working will be examined using binary and ordinal logistic regression analyses. Estimates of the proportion of HCWs likely to continue working will be determined after standardisation to the population of NHS employees.

## Ethical approval

National Research Ethics Service (NRES) and hospital Research and Development (R&D) approval are being applied for in two stages. Given that the content of the survey is dependent upon the phase one data, approval was first sought for phase one and for the general protocol for phase two. NRES approval for recruitment and running of focus groups, and distribution of questionnaires from Nottingham Research Ethics Committee 2 was granted on 29/10/07, and approval for the questionnaire was gained on 29/04/08 (ref. 07/H0408/120). R&D approval from participating Trusts was gained on 12/11/07, 17/12/07, and 18/12/07. Participating Trusts have not been named because doing so would compromise the anonymity of participants. Enquiries should be made to the corresponding author.

## Discussion

The threat of pandemic influenza is real and daunting, with predicted clinical attack and morbidity rates that could cause widespread social and economic disruption, placing enormous pressure on an already stretched NHS. The response of HCWs of all kinds to this pressure will determine how well the health service can cope during this crisis. Given the potential risks involved in working in a healthcare environment during an influenza pandemic, and the associated fears, it is vital to have information about how motivated HCWs will be to continue to work during such a crisis, and what factors might influence their decision to work/not to work. Through careful management of these factors it may be possible to implement strategies that will alleviate the concerns and fears of healthcare workers and remove potential barriers to working.

## Abbreviations

HCW: Healthcare worker; DH: Department of Health; WHO: World Health Organisation; NRES: National Research Ethics Service; NHS: National Health Service; NIHR: National Institute for Health Research; GP: General practitioner, HCA: Health care assistant.

## Competing interests

The authors declare that they have no competing interests.

## Authors' contributions

HD and SW drafted the study protocol with input from all authors and in collaboration with the Heart of England (Teaching) NHS Trust. JI wrote the first draft of the manuscript using the original protocol as a template, which has been commented on by the other authors. All the authors read and approved the final manuscript. Excepting the first three, authors are listed in alphabetical order.

## Pre-publication history

The pre-publication history for this paper can be accessed here:


